# Clinical epidemiology and predictors of outcome in children hospitalised with influenza A(H1N1)pdm09 in 2009: a prospective national study

**DOI:** 10.1111/irv.12286

**Published:** 2014-09-27

**Authors:** Gulam Khandaker, Yvonne Zurynski, Greta Ridley, Jim Buttery, Helen Marshall, Peter C Richmond, Jenny Royle, Michael Gold, Tony Walls, Bruce Whitehead, Peter McIntyre, Nicholas Wood, Robert Booy, Elizabeth J Elliott

**Affiliations:** a The Discipline of Paediatrics and Child Health, The University of SydneySydney, NSW, Australia; b National Centre for Immunisation Research and Surveillance of Vaccine Preventable Diseases, The Children's Hospital at WestmeadSydney, NSW, Australia; c The Marie Bashir Institute for Infectious Diseases and Biosecurity (MBI), Sydney Medical School, the University of SydneySydney, NSW, Australia; d The Australian Paediatric Surveillance UnitSydney, NSW, Australia; e Department of Paediatrics, Murdoch Children's Research Institute, Monash Children's Hospital, Monash UniversityMelbourne, Vic., Australia; f Vaccinology and Immunology Research Trials Unit, Women's and Children's Hospital, Robinson Institute and School of Paediatrics and Reproductive Health, University of AdelaideAdelaide, SA, Australia; g School of Paediatrics and Child Health, University of Western Australia, Princess Margaret Hospital for ChildrenPerth, WA, Australia; h Immunisation Service, Department of General Medicine, Royal Children's HospitalMelbourne, Vic., Australia; i School of Paediatrics and Reproductive Health, University of AdelaideAdelaide, SA, Australia; j The Sydney Children's Hospitals Network (SCHN)Sydney, NSW, Australia; k Department of Paediatrics, University of OtagoChristchurch, New Zealand; l Department of Paediatric Respiratory and Sleep Medicine, John Hunter Children's HospitalNewcastle, NSW, Australia

**Keywords:** Children, influenza, influenza A(H1N1)pdm09, outcome, pandemic

## Abstract

**Background:**

There are few large-scale, prospective studies of influenza A(H1N1)pdm09 in children that identify predictors of adverse outcomes.

**Objectives:**

We aimed to examine clinical epidemiology and predictors for adverse outcomes in children hospitalised with influenza A(H1N1)pdm09 in Australia.

**Methods:**

Active hospital surveillance in six tertiary paediatric referral centres (June–September, 2009). All children aged <15 years admitted with laboratory-confirmed influenza A(H1N1)pdm09 were studied.

**Results:**

Of 601 children admitted with laboratory-confirmed influenza, 506 (84·2%) had influenza A(H1N1)pdm09. Half (51·0%) of children with influenza A(H1N1)pdm09 were previously healthy. Hospital stay was longer in children with pre-existing condition (mean 6·9 versus 4·9 days; *P* = 0·02) as was paediatric intensive care unit (PICU) stay (7·0 versus 2·3 days; *P* = 0·005). Rapid diagnosis decreased both antibiotic use and length of hospital and PICU stay. Fifty (9·9%) children were admitted to a PICU, 30 (5·9%) required mechanical ventilation and 5 (0·9%) died. Laboratory-proven bacterial co-infection and chronic lung disease were significant independent predictors of PICU admission (OR 6·89, 95% CI 3·15–15·06 and OR 3·58, 95% CI 1·41–9·07, respectively) and requirement for ventilation (OR 5·61, 95% CI 2·2–14·28 and OR 5·18, 95% CI 1·8–14·86, respectively). Chronic neurological disease was a predictor of admission to PICU (OR 2·30, 95% CI 1·14–4·61).

**Conclusions:**

During the 2009 pandemic, influenza was a major cause of hospitalisation in tertiary paediatric hospitals. Co-infection and underlying chronic disease increased risk of PICU admission and/or ventilation. Half the children admitted were previously healthy, supporting a role for universal influenza vaccination in children.

*Please cite this paper as:* Khandaker *et al*. (2014) Clinical epidemiology and predictors of outcome in children hospitalised with influenza A(H1N1)pdm09 in 2009: a prospective national study. Influenza and Other Respiratory Viruses 8(6), 636–645.

## Background

The WHO declared the first influenza pandemic of the 21st century in June 2009.^1^ By the end of 2012, 18 449 deaths were reported worldwide due to influenza A(H1N1)pdm09 although this is likely a gross underestimation.^1^ Children are particularly vulnerable to seasonal or pandemic influenza, have the highest age-related hospitalisation rates and are the major community reservoir and source of infection.^2^ The 2009 pandemic had a significant impact on the paediatric population in terms of disease burden and severity.^3^,^4^

The epidemiology of the 2009 influenza pandemic in children was complex. Generally, it caused mild disease; however, many children required hospitalisation, some needed paediatric intensive care unit (PICU) admission and extracorporeal membrane oxygenation (ECMO) and many died.^5^,^6^ There are limited prospective, large-scale data on the overall burden of pandemic influenza hospitalisations, or predictors of severe and atypical complications and clinical outcomes in children hospitalised with influenza A(H1N1)pdm09. Reports from a variety of settings suggest an association between pre-existing neurological or respiratory conditions or chronic heart disease and poor outcomes.^7^–^10^

A recent report on influenza-associated intensive care unit (ICU) admissions and deaths in California, US between 29 September 2013 and 18 January 2014 identified influenza A (H1N1)pdm09 as the predominant circulating influenza virus this season. Among the 405 severe influenza cases (i.e. fatal and ICU cases combined), there were three fatal cases and 36 ICU cases among children aged <18 years. Most fatal cases (93%) had pre-existing medical conditions, and none had received 2013–2014 influenza vaccine. Moreover, over half of the hospitalised cases with fatal outcome did not receive antiviral treatment at presentation.^11^

In the 2013 Southern hemisphere influenza season, there has been a re-emergence of influenza A(H1N1)pdm09 virus, representing over 15% of overall notifications in Australia, compared to <1% of notifications in 2012. Both in 2012 and in 2013, about 12% of influenza cases have required ICU admission and a high proportion have comorbidities.^12^ Recent (as on 9 May 2014) Australian Influenza Surveillance Report and Activity Updates suggest that Australia is currently in the interseasonal period for influenza. Nationally, influenza A remains the predominant influenza virus type, of which A(H1N1)pdm09 is most common strain.^13^ Findings from our study are timely as we approach the 2014 influenza season in the Southern hemisphere. Here we aimed to examine the clinical epidemiology and predictors for a range of adverse outcomes in children hospitalised with pandemic influenza using national surveillance data prospectively collected in Australia.

## Methods

Active, hospital-based surveillance for cases of laboratory-confirmed influenza was performed in six tertiary/quaternary paediatric hospitals in Australia between 1 June 2009 and 30 September 2009 using the paediatric active enhanced disease Surveillance (PAEDS) mechanism, a collaborative project between the Australian Paediatric Surveillance Unit (APSU) and the National Centre for Immunisation Research and Surveillance (NCIRS). PAEDS is an inpatient surveillance system where specialist surveillance nurses actively ascertain cases of vaccine-preventable diseases, severe infection and adverse events following immunisation.^14^ Since 2007, four hospitals have participated in PAEDS: the Children's Hospital at Westmead (CHW); Royal Children's Hospital Melbourne (RCH); Women's and Children's Hospital Adelaide (WCH); and the Princess Margaret Hospital Perth (PMH). Influenza surveillance using PAEDS was made possible during the 2009 pandemic due to competitive emergency H1N1 funding from the National Health and Medical Research Council (NHMRC: Grant no: 633028) and additional funding from the NSW Department of Health. This funding also enabled inclusion of two additional hospitals, Sydney Children's Hospital (SCH) and John Hunter Children's Hospital (JHCH). During the study period, patients were identified from daily screening of virology results, emergency department presentations, new admissions and electronic inpatient databases; and contact with clinicians working in infectious diseases, intensive care, emergency, respiratory and general paediatrics.

### Participants

All children aged <15 years admitted with laboratory-confirmed influenza within the study period were eligible for inclusion. The PAEDS database included information on demographics, medical history, vaccination history, clinical presentation, diagnostic test results, treatment, clinical course and outcome.

### Diagnosis of influenza

Children presenting to surveillance hospitals with symptoms and/or signs consistent with influenza routinely had a nasopharyngeal aspirate or combined nose and throat swab collected for immunofluorescence and viral culture and/or PCR according to local hospital protocols. Many also had rapid antigen testing for influenza A and B as part of routine care. In 2009, specimens positive for influenza A were referred for virological characterisation to laboratories in each state, including the National Influenza Laboratory at Westmead Hospital in Sydney, Hunter Area Pathology Service (HAPS), South Eastern Area Laboratory Service (SEALS), Victorian Infectious Disease Reference Laboratory (VIDRL), SA Pathology, and PathWest Laboratory Medicine, Western Australia.

### Data collection

Data were collected using standardised protocols and questionnaires, entered onto local databases in each hospital and transferred to a central database in the APSU. Information was collected on demographic characteristics, past medical history, receipt of influenza vaccine and diagnostic tests. Clinical course, including presenting symptoms, complications associated with influenza infection, treatment and outcome, was documented. Outcome measures included length of hospital stay (LOS), requirement for and duration of PICU admission or ventilation, ongoing problems at discharge and 6 weeks after discharge, and death. Instances of nosocomial infection were defined as influenza-like symptoms commencing more than 48 hours after admission to hospital and later confirmed by a laboratory test. Data were collected prospectively from medical records, medical staff and family members, and the Australian Childhood Immunisation Register. Socio-economic Indexes for Areas (SEIFA) according to the child's postal code were determined.^15^ Parents/carers provided information to surveillance nurses via telephone on outcomes 6 weeks following discharge.

### Statistical analysis

Data analyses were performed using the Statistical Package for the Social Sciences (ibm spss® 19, Chicago, IL, USA). In addition to descriptive statistics, between-group proportions were compared using the chi-squared test with Yate's continuity correction for 2 × 2 tables and Pearson's correction for larger tables. Logistic regression analyses were conducted to identify independent predictors for key outcomes in children infected with pandemic influenza, namely (PICU) admission, requirement for ventilation, neurological complications and extended LOS (two or more days). Explanatory variables (including age at diagnosis, presence of any pre-existing chronic disease, chronic lung or neurological disease, immune compromise, vaccination, bacterial co-infection, presence of a smoker in the household and socio-economic status) were entered into a logistic regression model for each outcome in a forward stepwise manner in order of the strength of the univariate association and retained in the model if significant (*P* < 0·05). All explanatory variables included in the analyses are listed in Table 3, along with unadjusted (univariate) and adjusted (multivariate) odds ratios (ORs) and 95% confidence intervals (CI). Comparison of hospital and PICU admission rates was performed using StatXact.4.01 (StatXact Software; Cytel, Cambridge, MA, USA) to examine differences between two binomial populations.

**Table 3:** Predictors of PICU admission and requirement for ventilation in children with laboratory-confirmed H1N1 influenza (*n* = 506)

Explanatory variables	PICU Admission [*N* = 50/496]				Ventilated [*N* = 30/434]			
	Unadjusted OR (95% CI)	*P*-value	Adjusted OR (95% CI)	*P*-value	Unadjusted OR (95% CI)	*P*-value	Adjusted OR (95% CI)	*P*-value
Age < 6 months*	1·44 (0·64, 3·24)	0·51	–	–	1·64 (0·60, 4·49)	0·51	–	–
Age 6 months–4 years*	0·62 (0·34, 115)	0·17	–	–	0·67 (0·31, 1·43)	0·39	–	–
Pre-existing condition	1·91 (1·04, 3·50)	0·05	–	–	1·59 (0·73, 3·49)	0·33	–	–
Chronic neurological disease	3·25 (1·66, 6·35)	0·001	2·30 (1·14, 4·61)	0·02	2·61 (1·14, 5·98)	0·04	–	–
Chronic lung disease	6·31 (2·60, 15·30)	0·0001	3·58 (1·41, 9·07)	0·01	5·36 (1·95, 14·75)	0·001	5·18 (1·80, 14·86)	0·002
Immunocompromised	0·17 (0·02, 1·22)	0·08	0·14 (0·02, 1·06)	0·06	0·25 (0·03, 1·88)	0·25	–	
Bacterial co-infection**	3·69 (2·92, 3·98)	0·0001	6·89 (3·15, 15·06)	0·0001	5·76 (2·32, 14·28)	0·0001	5·61 (2·20, 14·28)	0·0001
Vaccination***	1·16 (0·49, 2·75)	0·92			1·74 (0·67, 4·53)	0·38	–	–
Smoker in household	1·40 (0·73, 2·72)	0·40			1·00 (0·42, 2·38)	1·00	–	–
SEIFA 1–3†	0·62 (0·21, 1·78)	0·51			0·44 (0·10, 1·92)	0·40	–	–

PICU, paediatric intensive care unit.

* Age ≥ 4 years (reference variable).

** Laboratory-proven bacterial co-infection at a sterile site within 72 hours of admission.

*** Vaccination for seasonal influenza.

† SEIFA (disadvantaged).

### Ethics approval

In NSW, the Department of Health approved immediate commencement of surveillance without prior ethics approval under provisions of the Health Records and Information Privacy Act (2002). Subsequent approval was obtained from the Human Research Ethics Committee at CHW and all participating hospitals.

## Results

### Characteristics of children identified by surveillance

**Figure 1:**
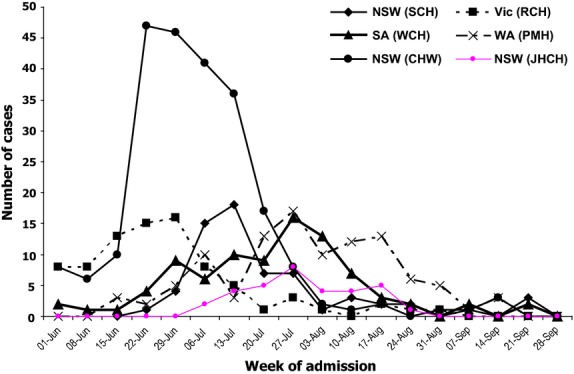
Timeline of admission in the participating hospitals.

During the surveillance period, 601 children were admitted to the six hospitals with laboratory-confirmed influenza, of whom 506 (84·2%) had influenza A(H1N1)pdm09 (Table 1). Admissions occurred between 1st June and 30th September 2009 with a peak in the fourth week of June (Figure 1). Based on total annual admissions to the six hospitals, the hospital admission rate for influenza was 33·9 per 1000 admissions in 2009. Of the 506 children with pandemic influenza, 58·9% were male and 93·3% were Australian born. The median age at admission was 3·7 years (range 0–14·9), with 22·2% aged <12 months and 56·5% <5 years. Almost a quarter (23·5%) reported living in a household with a current smoker.

**Table 1:** Demographics and clinical characteristics

Variables	*N* (%)
Number admitted with H1N1 09	506
Hospital admission rate/1000 hospital admissions*	33·9
Sex (Male)	298 (58·9)
Age in years: median (range)	3·7 (0–14·9)
Vaccinated for seasonal influenza in 2009	57 (11·3)
Households with a smoker	119 (23·5)
Ethnicity	
Caucasian	286 (56·5)
Middle-eastern	50 (9·9)
Asian	51 (10·1)
Aboriginal & Torres Strait Islander	23 (4·5)
Pacific Islander	17 (3·3)
Other (e.g. African)	79 (15·6)
Pre-existing conditions**	248 (49·1)
Chronic neurological disease	68 (13·4)
Immunocompromised	52 (10·3)
Asthma	51 (10·1)
Chronic lung disease (not asthma)	24 (4·7)
Chronic Heart disease	24 (4·7)
Chronic metabolic condition	18 (3·5)
Haemoglobinopathies	15 (2·9)
Diabetes	12 (2·4)

* Hospital admission rate = number of children aged <15 years admitted for laboratory-confirmed influenza compared to total number of children aged <15 years admitted to hospital for the period 1 June 2009–30 September 2009.

** One or more conditions.

The overall rate of seasonal influenza vaccination was 11·3% but varied by state (7·2% in NSW to 27·9% in WA). For children in whom trivalent inactivated influenza vaccine is recommended and funded under the Australian National Immunisation Program (i.e. children ≥6 months with an underlying chronic disease [*N* = 228]), only 42 (18·4%) reported having received at least one dose of seasonal influenza vaccine in 2009.

**Figure 2:**
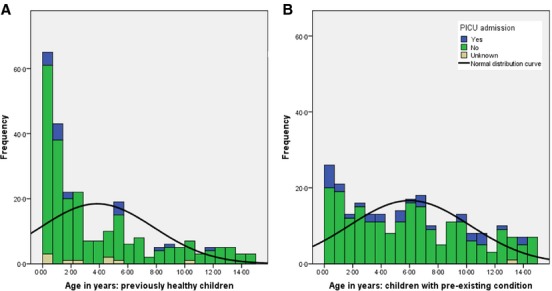
Age distribution of children with or without pre-existing condition and PICU admission.

Almost half of all children (*n* = 248; 49·1%) had at least one pre-existing chronic condition, most commonly chronic neurological disease, immune compromise or asthma. There was a significant difference in mean age between children with pre-existing medical conditions and without [6·0 (SD 4·24) versus 3·8 (SD 3·98) years; *P* = 0·005]. Most (90·8%) children acquired influenza infection in the community and in 68 (13·4%) contact with a person with an influenza-like illness was identified. There were 47 (9·2%) infections meeting the definition of nosocomial or hospital acquired, of which 38 (80·9%) occurred in chronically ill children who were being treated as inpatients for other disorders. Fifty (9·9%) children needed admission to a PICU, 30 (5·9%) required mechanical ventilation and 5 (0·9%) died of influenza or its complications. Figure 2 shows the age distribution of children with or without pre-existing conditions and PICU admissions.

### Presenting symptoms and clinical course

The most common presenting symptoms of pandemic influenza in children were cough (67·2%), fever (51·4%) and coryza (50·0%), but vomiting (32·2%) and diarrhoea (14·2%) were also reported frequently (Table 2). Cough, dyspnoea, sore throat and headache were more frequent at presentation in children with pre-existing conditions than in previously healthy children. Table 2 summarises presenting and clinical symptoms, complications, treatment and outcomes of the cohort.

**Table 2:** Clinical symptoms, complications, treatment and outcomes

	All cases (*n* = 506) *N* (%)	Pre-existing conditions (*n* = 248)	Previously healthy (*n* = 258)	*P*-value
Clinical symptoms				
Cough	340 (67·2)	181 (72·9)	159 (61·6)	**0·007**
Fever	260 (51·4)	138 (55·6)	122 (47·3)	0·060
Coryza	253 (50·0)	125 (50·4)	128 (49·6)	0·895
Malaise/lethargy	195 (38·5)	106 (42·7)	89 (34·5)	0·057
Vomiting	163 (32·2)	82 (33·1)	81 (31·4)	0·688
Dyspnoea	129 (25·5)	73 (29·4)	56 (21·7)	**0·046**
Diarrhoea	72 (14·2)	33 (13·3)	39 (15·1)	0·560
Sore throat	70 (13·8)	43 (17·3)	27 (10·5)	**0·025**
Headache	54 (10·7)	36 (14·5)	18 (6·9)	**0·006**
Seizure	33 (6·5)	20 (8·1)	13 (5·0)	0·168
Complications (any)	176 (34·8)	92 (37·1)	84 (32·5)	0·284
Pneumonia	90 (17·8)	45 (18·1)	45 (17·4)	0·836
Neurological complications (including seizure)	49 (9·7)	27 (10·9)	22 (8·5)	0·370
Bacterial co-infection	33 (6·5)	22 (8·9)	11 (4·3)	**0·036**
Viral co-infection	26 (5·1)	10 (4·0)	16 (6·2)	0·269
Seizure	17 (3·4)	6 (2·4)	11 (4·3)	0·250
Pleural effusion	9 (1·8)	5 (2·0)	4 (1·5)	0·692
ARDS	7 (1·4)	6 (2·4)	1 (0·4)	**0·050**
Encephalitis	7 (1·4)	3 (1·2)	4 (1·5)	0·743
POC tests	204 (40·3)	98 (39·5)	106 (48·1)	0·393
Treatment				
Oseltamivir	238 (47·0)	165 (66·5)	73 (28·3)	**0·000**
Zanamivir	3 (0·6)	2 (0·8)	1 (0·4)	–
Antibiotics	273 (54·0)	154 (62·1)	119 (46·1)	0·762
Outcomes				
Death	5 (0·9)	1 (0·4)	4 (1·5)	0·192
LOS in hospital: mean(SD) days	5·9 (10·8)	6·9 (10·6)	4·9 (10·9)	**0·026**
PICU admission	50 (9·9)	32 (12·9)	18 (6·9)	**0·005**
LOS in PICU: mean (SD) days	5·3 (6·4)	7·0 (7·2)	2·3 (2·5)	**0·005**
Ventilated	30 (5·9)	20 (8·1)	10 (3·9)	0·058
Duration of ventilation: mean (SD) days	6·8 (11·7)	7·5 (12·5)	5·5 (10·5)	0·888

ARDS, Acute respiratory distress syndrome; LOS, length of hospital stay; SD, standard deviation; POC, Point of care tests (rapid antigen test).

Bold values indicates significant difference between groups.

### Complications and uncommon syndromes associated with pandemic influenza

In this large prospective national surveillance study, we identified many atypical or uncommon presenting symptoms of pandemic influenza. Rash is not commonly reported in influenza, but we identified 31 children (6·1%) with a non-specific rash at presentation to hospital. Other uncommonly reported symptoms at presentation or during admission included difficulty walking (39, 7·7%), loss of consciousness (*n* = 5), apnoea (*n* = 2), weakness (*n* = 1) and pallor with jaundice (*n* = 1).

Specific complications associated with influenza infection were detected in 176 (34·8%) children; most commonly X-ray-proven pneumonia in 90 (17·8%). Neurological complications occurred in 49 (9·7%), primarily seizures on presentation or during admission in 38 (77·5%). Laboratory-proven bacterial co-infection in a sterile site/blood culture was detected in 29 (5·7%) children. Organisms isolated included *Pseudomonas aeruginosa* (*n* = 5), *Streptococcus pneumoniae* (*n* = 5), *Proteus* sp. (*n* = 3), non-type b *Haeomophilus influenzae* (*n* = 3), *Coagulase-negative staphylococci* (*n* = 3), *Staphylococcus aureus* (*n* = 2), *Neisseria meningitidis* (*n* = 2), *Escherichia coli* (*n* = 2)*, Enterobacter* (*n* = 2), *Moraxella catarrhalis* (*n* = 1) *and Micrococcus* (*n* = 1). *Bordetella pertussis* was isolated in respiratory specimens in four children. Laboratory-proven viral co-infection was evident in 26 (5·1%) children, most commonly respiratory syncytial virus (*n* = 11) and adenovirus (*n* = 4).

Several children in our cohort had atypical and rare complications of pandemic influenza, including seven cases of encephalopathy/encephalitis, three cases of cardiomyopathy and two cases of cardiac arrest (child had bradycardic arrest due to hypoxia from right upper lung collapse, and the other had ventricular fibrillation arrest associated with long QT syndrome and hypertrophic cardiomyopathy). Two cases of acute renal failure included one with co-infection with Epstein–Barr virus and one with two relapse of thrombotic thrombocytopenic purpura. There was one case each of stroke, Guillain-Barre syndrome, transverse myelitis, myocarditis, pericarditis and rhabdomyolysis.

### Clinical management and outcomes

Over half of all children (54·0%) received antibiotics, and almost half (47%) were treated with oseltamivir. Mean duration of influenza symptoms prior to the commencement of oseltamivir treatment was 4·1 days (median 2 days). Oseltamivir was used more commonly in children with pre-existing medical conditions than in previously healthy children (66·5% versus 28·3%; *P* = 0·0001). Under the provision of emergency use authorisation,^16^ 47 infants (42% of all infants) were treated with oseltamivir. Of these, 28 were aged <6 months and 16 had a pre-existing condition. There was no significant difference in the LOS of infants treated with oseltamivir compared with non-treated infants (mean 6·7 days versus 5·5 days, *P* = 0·87). There were no significant differences in rates of vomiting (*P* = 0·09) and seizures (*P* = 0·30) at presentation, later development of seizures (*P* = 0·55) or any neurological complication (*P* = 0·61) in infants treated with oseltamivir or not. In one child, the influenza A(H1N1)pdm09 isolate was resistant to oseltamivir (275Y mutant).

The mean LOS was 5·9 days (median 2 days, range 1–107 days) for all children who acquired influenza infection in the community, compared with 13·4 days (median 10 days, range 1–50 days) for children with nosocomial infection, most of whom (*n* = 29) had pre-existing conditions. The mean length of PICU stay for all children was 5·3 days (median 2 days, range 1–30 days). Children with pre-existing conditions were more often admitted to PICU (12·9% versus 6·9%, *P* = 0·005) and had a longer duration of PICU stay (7·2 days versus 2·5 days *P* = 0·005) compared to previously healthy children. One child with Burkitt's lymphoma and co-infection with invasive Streptococcus type A infection required ECMO.

Telephone follow-up was conducted 6 weeks after discharge from hospital. Influenza-like symptoms at discharge were reported by 116 (23·4%) parents and included cough, lethargy, fever, rhinorrhoea, poor appetite and abnormal lung function (in children with known chronic lung disease). Parents of 67 (13·5%) children reported ongoing problems 6 weeks after discharge, including persistent cough (*n* = 14), bronchitis and breathing difficulties (*n* = 10), influenza-like symptoms (*n* = 4), lethargy (*n* = 4) and rhinorrhoea (*n* = 3). Of these 38 (56·7%) had a pre-existing condition.

### Rapid diagnostic test and outcomes

In our cohort, 204 children (40·3%) had a rapid antigen test on presentation to hospital, and these children had a significantly lower rate of antibiotic treatment (57·8% versus 68·5%, *P* = 0·03) and shorter hospital (mean 4·3 versus 7·3 days, *P* = 0·001) and PICU (mean 3·5 versus 5·2 days, *P* = 0·02) stay compared with children not tested. Oseltamivir treatment was commenced earlier in those who had a rapid antigen test than others (mean time between symptom onset and commencement of oseltamivir, 3·7 days versus 4·4 days, *P* = 0·34). There was no significant difference in the use of rapid antigen testing in children with or without pre-existing conditions (*P* = 0·39).

### Fatal cases

In our study, five children died due to influenza A(H1N1)pdm09 infection and its complications. All but one had a chronic underlying medical condition (two had epilepsy and developmental delay; one had trisomy 21, epilepsy and chronic kidney disease; and one immunocompromised child had a brainstem glioblastoma). Among the fatal cases, four children were male and their ages ranged from 4 to 12 years. Three of the five children who died were treated with oseltamivir, of whom two were admitted to PICU.

### Prediction of outcome

Table 3 shows the unadjusted (univariate) and adjusted (multivariate) odds ratios (ORs) for a range of predictors of PICU admission and requirement for ventilation. Laboratory-proven bacterial co-infection and chronic lung disease were significant independent predictors of PICU admission (OR 6·89, 95% CI 3·15–15·06 and OR 3·58, 95% CI 1·41–9·07) and requirement for ventilation (OR 5·61, 95% CI 2·2–14·28 and OR 5·18, 95% CI 1·8–14·86). Chronic neurological disease was a significant, but weaker predictor of admission to PICU (OR 2·30, 95% CI 1·14–4·61). The risk of admission to PICU is likely to be even higher for children with a combination of the significant predictors identified in the logistic regression model, for example, of eight children admitted to hospital with pre-existing neurological disease and bacterial co-infection, five were admitted to PICU, and of three children with chronic lung disease and chronic neurological disease and bacterial co-infection, two were admitted to PICU. Due to the small size of these subgroups, combined OR were not calculated. Immune compromise was not a significant predictor of PICU admission (OR 0·14; CI 0·02–1·06), and only one of the 52 children who were immunocompromised was admitted to PICU.

## Discussion

We used a novel, active, inpatient paediatric surveillance system i.e. PAEDS to prospectively collect one of the largest cohorts of laboratory-confirmed pandemic influenza in children. In our study, we report only cases of severe pandemic influenza, that is cases requiring hospitalisation, and include important information on clinical management and predictors of severe outcomes.

For pandemic preparedness, it is crucial to understand predictors of adverse outcomes because these inform the need for escalated medical services. We identified that laboratory-proven bacterial co-infection and chronic lung disease are significant independent predictors of PICU admission and requirement for ventilation. Chronic neurological disease is a significant but weaker predictor of admission to PICU. These findings are consistent with the recent literature^7^–^9^,^17^ and suggest the need for more targeted prevention and management strategies during pandemic and seasonal influenza epidemics.

Consistent with other studies, we report a high burden of paediatric hospitalisation due to pandemic influenza in 2009 (33·9 per 1000 admission in 2009).^4^,^18^ One in ten children hospitalised with pandemic influenza in 2009 required PICU admission, a rate similar to that reported at SCHN for seasonal influenza in 2003 (8·6%) and 2007 (10·1%).^19^ Consistent with seasonal influenza studies, most hospitalisations in 2009 were in young children (median age 3·7 years) and almost a quarter (22·2%) were aged <12 months.^19^

Universal seasonal influenza vaccination is not recommended in Australia; however, in 2009, a publicly funded programme was introduced to vaccinate all children aged between 6 months and 5 years in WA, accounting for the higher overall vaccination rate in WA than in the remaining states in all children (27·9% versus 9·3%) and in children aged 6 months to 5 years (30·3% versus 7·5%, respectively). Consistent with reports on seasonal influenza in children, almost half of the children (49·0%) in our cohort had one or more pre-existing medical conditions and thus were at higher risk of influenza-related complications.^19^ In Australia, seasonal influenza vaccine is recommended and publicly funded for children aged 6 months and over who have specific underlying medical conditions.^20^ In our cohort, seasonal influenza vaccine uptake was low among children at risk (18·4%). Notably, 51·0% of children hospitalised with severe influenza in this study were previously healthy children who are ineligible for influenza vaccination on the National Immunisation Program.

In this large prospective national surveillance study, we identified both common and rare clinical presentations and complications of pandemic influenza in children. Interestingly, the typical constitutional symptoms of influenza (cough, runny nose and fever) were absent in almost one-third of children. There were significant differences in clinical symptoms between children with underlying medical conditions and previously healthy children: cough, fever, malaise/lethargy, dyspnoea, sore throat and headache were more common in children with pre-existing chronic conditions. Some of these differences may relate to the older age of the children with pre-existing conditions and hence their ability to articulate symptoms. Rare presenting symptoms of influenza observed included neuromuscular weakness, pallor with jaundice and VF arrest. Clinicians should be watchful for these symptoms during future outbreaks of seasonal and pandemic influenza.

One-third of the children (34·8%) in our study developed one or more complications related to pandemic influenza. Respiratory complications (20·8%) and neurological complications (10·4%) occurred more frequently than during seasonal influenza epidemics.^21^ In this large study, we were also able to capture some of the rare complications of pandemic influenza in children, including stroke, Guillain-Barre Syndrome, transverse myelitis (TM), myocarditis, pericarditis and rhabdomyolysis. There have been a few case reports of these atypical and rare complications of pandemic influenza, consistent with our findings.^22^–^24^ However, to date, no study has reported so many rare complications of pandemic influenza in a single national cohort of children. There have been a few reports of TM following influenza vaccination (especially the pandemic H1N1 vaccine)^25^; however, in our study, one unvaccinated child developed TM as a complication of pandemic influenza.

Oseltamivir was used in Australia more widely in 2009 than during previous seasonal epidemics of influenza.^26^ In our large cohort, we were unable to show any significant benefit of oseltamivir in reducing the LOS. However, a significantly higher proportion of both children with underlying medical conditions and children who were admitted to PICU were treated with oseltamivir, which is likely to be a confounding factor in this analysis. Oseltamivir was only used after hospital admission, and the duration between symptom onset and commencement of oseltamivir therapy often exceeded 72 hours. Although oseltamivir treatment was not approved for use in infants aged <12 months, during the 2009 pandemic, the US Food and Drug Administration (FDA) approved therapeutic and prophylactic oseltamivir use in infants aged <1 year.^16^ The Centres for Disease Control and Prevention (CDC) and the American Academy of Pediatrics currently recommend therapeutic use of oseltamivir for infants <1 year of age.^27^ In our cohort, 47 infants (42% of all infants) were treated with oseltamivir. Oseltamivir was well tolerated in this group without any adverse event. To date, ours is one of the largest cohorts of infants treated with oseltamivir during the 2009 pandemic.

Conventional diagnostic tests for influenza (e.g. PCR, IF, culture) are time consuming and expensive. However, rapid antigen tests (point of care tests) are quick and highly sensitive and specific if properly performed in children.^28^ Rapid antigen tests were performed more frequently during the 2009 pandemic than in previous seasonal influenza outbreaks.^26^ In our cohort, 40·3% had a rapid antigen test on presentation to hospital. Children who had a rapid antigen test on presentation required significantly reduced length of PICU and hospital stay compared with others. Moreover, Oseltamivir treatment was commenced earlier in those who had a rapid antigen test than others (mean time between symptom onset and commencement of oseltamivir, 3·7 days versus 4·4 days) which could partially explain the reduced length of PICU and hospital stay in this group. Our findings strongly support the utility of rapid antigen testing and its wider use in paediatric emergency settings during influenza epidemics and future pandemics.

## Conclusions

In one of the largest reported cohorts of children hospitalised during the 2009 pandemic, we confirm that influenza and its complications are a major cause of paediatric hospitalisation in tertiary paediatric hospitals in Australia. Laboratory-proven bacterial co-infection, chronic lung disease and chronic neurological disease should alert clinicians to a higher risk of PICU admission and/or requirement for ventilation. The role of rapid diagnosis and oseltamivir in improving outcomes in infants and children with influenza requires further investigation. Importantly, we show that previously healthy children are vulnerable to severe influenza and its complications, supporting universal seasonal influenza vaccination.

## Author contributions

Gulam Khandaker (gulam.khandaker@health.nsw.gov.au): involved in data analysis and interpretation and contributed to the first and subsequent drafts of the manuscript. Yvonne Zurynski (yvonne.zurynski@health.nsw.gov.au): contributed to development of the initial concept, protocols and data collection forms; data analysis and critical review of the first and subsequent version of the manuscript. Greta Ridley (greta.ridley@health.nsw.gov.au): involved in data analysis and interpretation and contributed to the first and subsequent drafts of the manuscript. Jim Buttery (jim.buttery@mcri.edu.au): contributed to collection of surveillance data, data cleaning, study analysis and drafting the manuscript and approved the final version to be submitted. Helen Marshall (helen.marshall@adelaide.edu.au): contributed to collection of surveillance data, data cleaning, study analysis and drafting the manuscript and approved the final version to be submitted. Peter C Richmond (prichmond@meddent.uwa.edu.au): contributed to collection of surveillance data, data cleaning, study analysis and drafting the manuscript and approved the final version to be submitted. Jenny Royle (jenny.royle@bigpond.com): contributed to collection of surveillance data, data cleaning, study analysis and drafting the manuscript and approved the final version to be submitted. Michael Gold (michael.gold@adelaide.edu.au): contributed to collection of surveillance data, data cleaning, study analysis and drafting the manuscript and approved the final version to be submitted. Tony Walls (tony.walls@otago.ac.nz): contributed to collection of surveillance data, data cleaning, study analysis and drafting the manuscript and approved the final version to be submitted. Bruce Whitehead (Bruce.Whitehead@hnehealth.nsw.gov.au): contributed to collection of surveillance data, data cleaning, study analysis and drafting the manuscript and approved the final version to be submitted. Peter McIntyre (peter.mcintyre@health.nsw.gov.au): contributed to collection of surveillance data, data cleaning, study analysis and drafting the manuscript and approved the final version to be submitted. Nicholas Wood (nicholas.wood@health.nsw.gov.au): contributed to collection of surveillance data, data cleaning, study analysis and drafting the manuscript and approved the final version to be submitted. Robert Booy (robert.booy@health.nsw.gov.au): liaised with additional hospitals in NSW to obtain their involvement in the study. Contributed to development of the initial study concept, protocols and data collection forms; data analysis and critical review of the first and subsequent version of the manuscript. Elizabeth J Elliott, (elizabeth.elliott@health.nsw.gov.au): liaised with additional hospitals in NSW to obtain their involvement in the study. Contributed to development of the initial study concept, protocols and data collection forms; data analysis and critical review of the first and subsequent version of the manuscript.

## References

[1] World Health Organization http://www.who.int/csr/don/2010_08_06/en/index.html.

[2] Carr S (2012). Seasonal and pandemic influenza: an overview with pediatric focus. Adv Pediatr.

[3] Elliott EJ, Zurynski YA, Walls T (2012). Novel inpatient surveillance in tertiary paediatric hospitals in New South Wales illustrates impact of first-wave pandemic influenza A H1N1 (2009) and informs future health service planning. J Paediatr Child Health.

[4] Libster R, Bugna J, Coviello S (2010). Pediatric hospitalizations associated with 2009 pandemic influenza A (H1N1) in Argentina. N Engl J Med.

[5] Yung M, Slater A, Festa M (2011). Pandemic H1N1 in children requiring intensive care in Australia and New Zealand during winter 2009. Pediatrics.

[6] Morgan CI, Hobson MJ, Seger B (2012). 2009 Pandemic influenza A (H1N1) in critically ill children in Cincinnati, Ohio. Pediatr Crit Care Med.

[7] Mertz D, Kim TH, Johnstone J (2013). Populations at risk for severe or complicated influenza illness: systematic review and meta-analysis. BMJ.

[8] Chong CY, Tan NW, Menon A, Thoon KC, Tee NW, Fu S (2013). Risk factors for complicated influenza A (H1N1) 2009 disease in children. Ann Acad Med Singapore.

[9] Myles PR, Semple MG, Lim WS (2012). Predictors of clinical outcome in a national hospitalised cohort across both waves of the influenza A/H1N1 pandemic 2009–2010 in the UK. Thorax.

[10] Goenka A, Michael BD, Ledger E (2014). Neurological manifestations of influenza infection in children and adults: results of a National British Surveillance Study. Clin Infect Dis.

[11] Ayscue P, Murray E, Uyeki T (2014). Influenza-associated intensive-care unit admissions and deaths - California, September 29, 2013–January 18, 2014. MMWR Morb Mortal Wkly Rep.

[12] http://www.health.gov.au/internet/main/publishing.nsf/Content/cda-ozflu-no09-13.htm.

[13] http://www.health.gov.au/internet/main/publishing.nsf/Content/cda-surveil-ozflu-flucurr.htm.

[14] Zurynski Y, McIntyre P, Booy R, Elliott EJ, PAEDS Investigators Group (2013). Paediatric active enhanced disease surveillance: a new surveillance system for Australia. J Paediatr Child Health.

[15] Australian Bureau of Statistics

[16] United States Food and Drug Administration (FDA) http://www.fda.gov/downloads/.

[17] Khandaker G, Zurynski Y, Buttery J (2012). Neurologic complications of influenza A(H1N1)pdm09: surveillance in 6 pediatric hospitals. Neurology.

[18] Da Dalt L, Chillemi C, Cavicchiolo ME (2011). Pandemic influenza A (H1N1v) infection in pediatric population: a multicenter study in a North-East area of Italy. Ital J Pediatr.

[19] Khandaker G, Lester-Smith D, Zurynski Y, Elliott EJ, Booy R (2011). Pandemic (H1N1) 2009 and seasonal influenza A (H3N2) in children's hospital, Australia. Emerg Infect Dis.

[20] National Health and Medical Research Council (2008). The Australian Immunisation Handbook.

[21] Ekstrand JJ, Herbener A, Rawlings J (2010). Heightened neurologic complications in children with pandemic H1N1 influenza. Ann Neurol.

[22] Honorat R, Tison C, Sevely A, Cheuret E, Chaix Y, Claudet I (2012). Influenza A(H1N1)-associated ischemic stroke in a 9-month-old child. Pediatr Emerg Care.

[23] Jang JY, Chang HJ, Jang Y (2010). Constrictive pericarditis accompanied by swine-origin influenza A (H1N1) infection. Korean Circ J.

[24] Unverdi S, Akay H, Ceri M (2011). Acute kidney injury due to rhabdomyolysis in H1N1 influenza infection. Ren Fail.

[25] Gui L, Chen K, Zhang Y (2011). Acute transverse myelitis following vaccination against H1N1 influenza: a case report. Int J Clin Exp Pathol.

[26] Lester-Smith D, Zurynski YA, Booy R (2009). The burden of childhood influenza in a tertiary paediatric setting. Commun Dis Intell.

[27] Centers for Disease Control and Prevention, Influenza Antiviral Medications: Summary for Clinicians http://www.cdc.gov/flu/professionals/antivirals/summary-clinicians.htm.

[28] Andresen DN, Kesson AM (2010). High sensitivity of a rapid immunochromatographic test for detection of influenza A virus 2009 H1N1 in nasopharyngeal aspirates from young children. J Clin Microbiol.

